# Epidemiologic and clinical characteristics of human bocavirus infection in infants and young children suffering with community acquired pneumonia in Ningxia, China

**DOI:** 10.1186/s12985-021-01682-1

**Published:** 2021-10-29

**Authors:** Kai Ji, Jinhan Sun, Yan Yan, Lei Han, Jianhui Guo, Anwen Ma, Xueqi Hao, Fang Li, Yuning Sun

**Affiliations:** 1 Department of Biochemistry and Molecular Biology, Key Laboratory of Fertility Preservation and Maintenance of Ministry of Education, School of Basic Medical Science, Ningxia Medical University, Yinchuan, 750004 China; 2 School of Clinical Medicine, Hainan Medical University, Haikou, 571199 China; 3 Department of Respiratory and Critical Care, General Hospital of Ningxia Medical University, Yinchuan, 750004 China; 4 Department of Clinical Laboratory, Yinchuan Women and Children Healthcare Hospital, Yinchuan, 750001 China; 5 Department of Clinical Medicine, Ningxia Medical University, Yinchuan, 750004 China

**Keywords:** Community acquired pneumonia, Human bocavirus, Co-infection, Bacteria, *Mycoplasma pneumoniae*

## Abstract

**Background:**

Pneumonia has a high incidence rate and is a major cause of mortality in children, mostly community-acquired pneumonia (CAP). Human bocavirus (HBoV), since it first identified in 2005, has been repeatedly associated with respiratory tract infections. Nevertheless, the role and related information of HBoV as a pathogen of CAP has not been fulfilled. Here our study is to assess the epidemiological and clinical features in HBoV-positive children with CAP.

**Methods:**

A total of 878 secretions of lower respiratory samples were obtained, multiplex PCR was used to detect HBoV and other respiratory viruses.

**Results:**

Of all cases, HBoV was detected in 10.0%, with a peak incidence of infection among children < 2 year old, and predominantly noted in autumn and winter. Only 8 patients were HBoV single infection. Co-infection with other respiratory viruses was observed in 86.4%. Moreover, co-infection with bacteria occurred in 27.3% and with *Mycoplasma* *pneumoniae* (MP) in 33.0% of HBoV-positive patients. Among all HBoV-positive samples co-infected with bacteria, 87.5% are gram negative bacteria. Compared with HBoV-negative group, age (*P* = 0.048), wheezing (*P* = 0.015), tachypnea (*P* = 0.016), lactate dehydrogenase (*P* = 0.026) and severe pneumonia (*P* = 0.023) were statistically significant in HBoV-positive patients. Furthermore, HBoV-positive patients less than 1 year old were more likely to have co-infection with bacteria (*P* = 0.007).

**Conclusions:**

HBoV can be detected alone in respiratory samples of children with CAP, maybe it is one of the causes of CAP in infants. The high incidence of severe pneumonia was found in HBoV-positive patients compared with HBoV-negative cases may indicate a relationship between severe pneumonia and HBoV.

## Background

Pneumonia is a frequently diagnosed disease, mostly CAP, for hospitalization and a major cause of mortality in infants and children worldwide, especially in developing countries [1, 2]. Multiple pathogens are capable of causing CAP, viruses are the main pathogens for it. Previous investigations of CAP in children have shown that respiratory syncytial virus (RSV), influenza virus (IFV), human rhinovirus (HRV) were identified as common causes of viral CAP [3–5]. Since HBoV first identified in 2005 [6], it has been repeatedly detected in respiratory tract infections [7]. Nevertheless, the process that HBoV talks with host cells as a pathogen of CAP is unclear, mainly due to the lack of specific cell lines for virus culture or experimental animal models [8].

HBoV is small non-enveloped single-stranded DNA viruses of the *Parvoviridae* family with four HBoV genotypes, HBoV-1 has been mainly identified in respiratory samples, whereas genotypes 2 to 4 of HBoV are principally detected in intestinal infection [9]. It has been reported with frequencies ranging from 1.9 to 24.6% in respiratory samples [10], mostly from children with acute respiratory tract infection, however, HBoV can also be detected in asymptomatic people [8], Probably because HBoV-1 has been shown to stay in the nasopharynx for weeks and even months after acute infection, thereby posing a challenge to diagnosis of acute HBoV-1 infection [11]. HBoV can exist alone or be detected with other pathogens, however latter is more common [12]. Interestingly, recent data suggest that HBoV as the single causative agent also caused severe acute respiratory tract infection [4], and our research also support this conclusion. By now, the information about the epidemiological and clinical characteristics of CAP caused by HBoV in infants and young children is limited. In this study, we described the prevalence of HBoV in infants and children who presented at the hospital with CAP, the clinical features of the infected children, and a phylogenetic analysis of HBoV was also carried out.

## Materials and methods

### Patients and clinical assessment

A total of 878 children who were hospitalized with CAP and less than 14 years of age at pediatric department of Yinchuan Women and Children Healthcare Hospital in 2018 and 2019 were enrolled in study. All patients were diagnosed according to WHO’s clinical criteria of CAP including respiratory symptoms and chest radiographic findings as evaluated by attending physicians. As part of the research, study questionnaire with the following variables: age, sex, month of admission, diagnosis, clinical symptoms, physical examination and laboratory examinations on admission were recorded.

### Specimen collection

Secretions of lower respiratory tract were obtained by suction using a fine, flexible plastic catheter, and then immediately diluted (the ratio of 1 to 1) with virus protection solution and stored at − 80 °C before use. One sample was collected from each patient.

### Detection of HBoV and other viruses

**Fig. 1:**
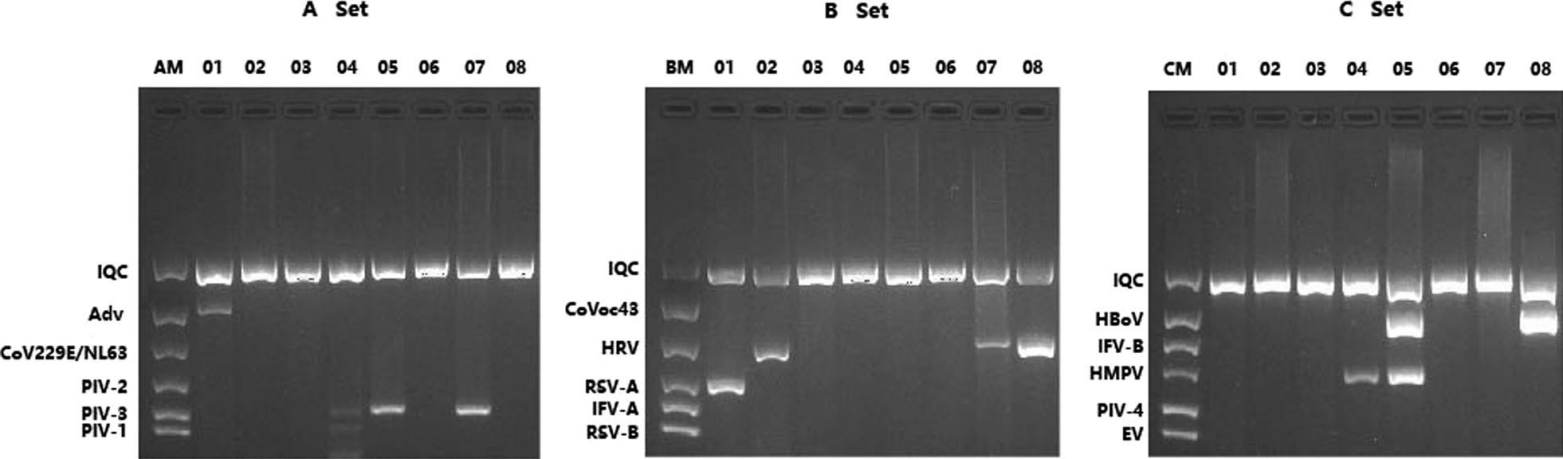
Results of PCR products electrophoresis. There are 8 samples, A Set, B Set and C Set showed the detection of viruses in 8 samples. No. 01 were positive for AdV and RSV-A; No. 02 was positive for HRV; No. 03 was negative for viruses; No. 04 were positive for PIV-1, PIV-3 and HMPV; No. 05 were positive for HBoV, PIV-3 and HMPV; No. 06 shows no virus; No. 07 were positive for HRV and PIV-3; No. 08 were positive for HBoV and HRV. AM, A marker; BM, B marker; CM, C marker; IQC, internal quality control

The detection of viruses involves the following steps, extraction of DNA/RNA from samples, reverse transcription and multiplex PCR. Viral nucleic acid was extracted from 100ul sample solution using a Quick DNA/RNA Viral Kit (Zymo Research, America) according to the manufacturer’s instructions, this kit enables simultaneous extraction of viral RNA and DNA. Then, cDNA was synthesized by reverse transcription kit (Transgen, China). We detected viruses using multiplex PCR kit (Seegene, Korea).There are three groups of kit (Fig. 1). A set includes adenovirus (AdV), human coronavirus (HCoV) 229E/NL63 and PIV-1 to 3 (parainfluenza virus types 1, 2 and 3). B set includes human CoVoc43, RSV A/B, HRV and IFV-A. Human metapneumovirus (HMPV), IFV-B (Influenza B virus), PIV-4 (parainfluenza virus types 4), HBoV and Enterovirus (EV) are included in C set. The products of PCR were identified by gel electrophoresis.

### Detection of respiratory bacteria

All lower respiratory tract specimens from patients were routinely submitted to the clinical laboratory of the pediatric department of Yinchuan Women and Children Healthcare Hospital. Specimens were planted on blood-, chocolate- (with vancomycin), MacConkey-agar plate for bacteria cultures.

### Detection of MP

Venous blood was collected from children, and centrifuged to separate the serum, and the antibody against MP was detected by passive particle agglutination kit (SERODIA®-MYCO II), all specimens were tested in accordance with the methods specified in the kit, single serum antibody titer ≧ 1:40 indicates positive infection.

### Phylogenetic analysis

We selected eight HBoV-positive samples and sequenced partial fragments of NS1 (600 bp). The nucleotide sequences of the NS1 gene were compared with other sequences available in GenBank (including HBoV reference strains BLR/Minsk/11/14, BLR/Minsk/10/14, ST1, ST2, JX2239, YOK/08/104, CU6, BLR/Gomel/285/15, BLR/Mogilev/241/14, USA/UNM-0238, USA/TCNP-0070, Eg/BSU-1, Eg/BSU-2, Eg/BSU-3, Mty117, BRA/TO-40, BRA/TO-57, BRA/TO-243, BRA/TO-237, Canine minute virus and Human parvovirus B19). Phylogenetic tree with 1,000 bootstrap replicates was generated using the maximum likelihood method with MEGA 7.0 software.

### Statistical analysis

Analyses were conducted using SPSS 22.0. Measurement data do not accorded with normal distribution were expressed as median (IQR), and data were compared by rank sum test. Categorical variables were expressed as numbers or percentages, proportion were compared by chi-squared.Two-sided *P* values < 0.05 were considered statistically significant.

## Results

### Characteristics of patients

We collected a total of 878 samples of the respiratory tract secretions from children with CAP. The male (n = 510) to female (n = 368) ratio was 1.4:1, and the median age was 30 months (age range = less than 1 month to 13 years 5 months). 52.6% of patient were aged < 2, 33.8% aged 2 -5 and 13.6% aged ≧ 5 years.

### Detection of pathogens

**Fig. 2:**
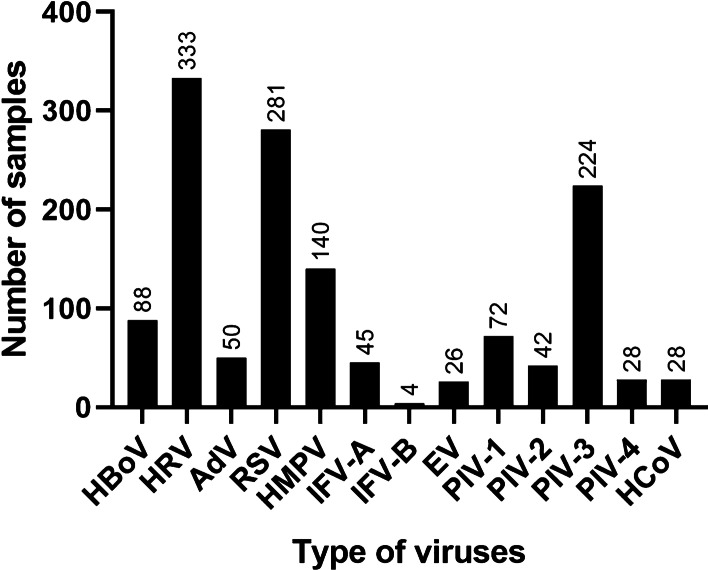
The detection of respiratory viruses

Of the 878 patients with CAP, virus was positive in 749 (85.3%) patients, bacteria in 217 (24.7%) patients, and MP in 184 (21%) patients (Table 1). HBoV was detected in 88 samples (10.0%), and detection rate of other viruses (in descending order) were as follows (Fig. 2): HRV (n = 333, 37.9%), RSV (n = 281, 32.0%), PIV-3 (n = 224, 25.5%), HMPV (n = 140, 15.9%), PIV-1 (n = 72, 8.2%), AdV (n = 50, 5.7%), IFV-A (n = 45, 5.1%), PIV-2 (n = 42, 4.8%), PIV-4 (n = 28, 3.2%) and HCoV (n = 28, 3.2%), EV (n = 26, 3.0%), IFV-B (n = 4, 0.5%).

**Table 1:** The detection of pathogen in all samples

Variable	n (%)
	n = 878
Virus-positive	749 (85.3%)
Bacterial-positive	217 (24.7%)
*Mycoplasma*-positive	184 (21.0%)
HBoV-positive	88 (10.0%)
	n = 88
HBoV only	8 (9.1%)
HBoV + other viruses	76 (86.4%)
HBoV + bacteria	24 (27.3%)
HBoV + MP	29 (33.0%)

### Viral seasonal distribution of HBoV

**Fig. 3:**
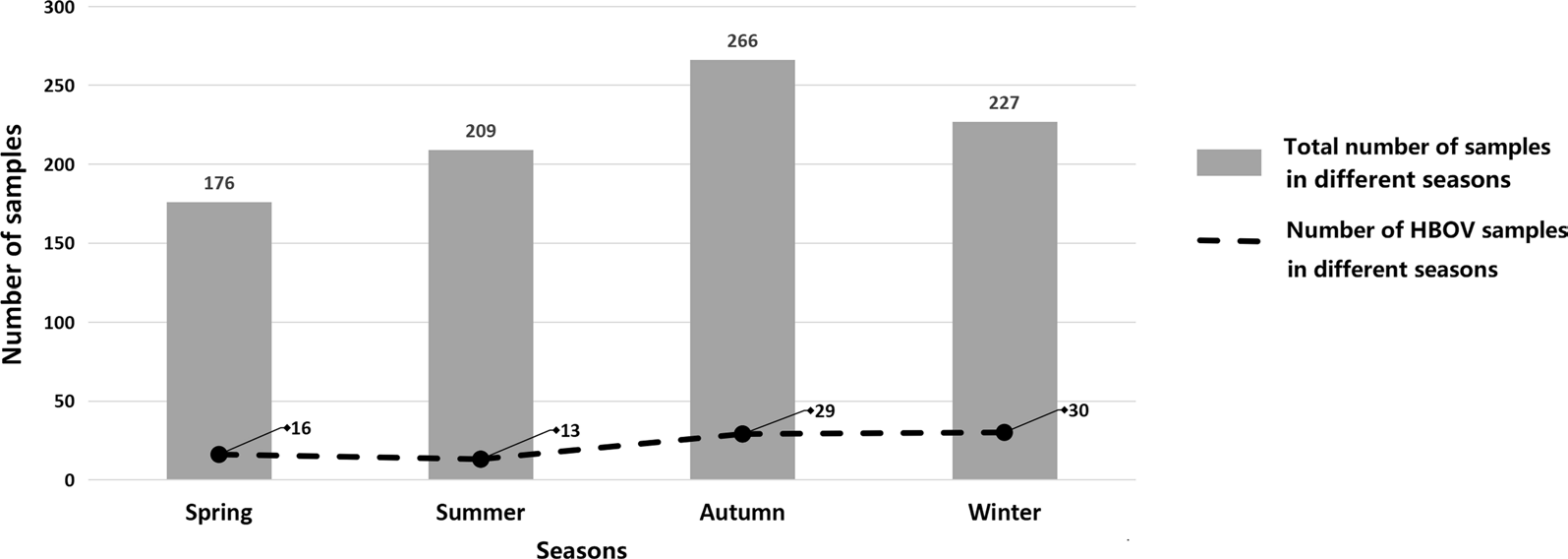
Viral seasonal distribution of HBoV. Spring: February to April; Summer: May to July; Autumn: August to October; Winter: November to January

The seasonal distributions of all patients with HBoV infections in 2018 and 2019 were shown in Fig. 3, HBoV infection were detected in each season with a peak incidence in winter (34.1%), followed by autumn (33.0%).

### Clinical characteristics of patients with or without HBoV

Patients were divided into two groups (Table 2). Among the 88 HBoV-positive patients and 790 HBoV-negative patients, 57 (64.8%) versus 453 (57.3%) were male and 31 (35.2%) versus 337 (42.7%) were female, no significant difference in gender between two groups (*P* = 0.180).

**Table 2:** Clinical characteristics of HBoV-positive and HBoV-negative patients

Variable	HBoV-positive (n = 88)	HBoV-negative (n = 790)	X^2^/Z	*P*
Gender			1.796	0.180
Male	57 (64.8%)	453 (57.3%)		
Female	31 (35.2%)	337 (42.7%)		
Age			6.059	0.048*
< 2	56 (63.6%)	406 (51.4%)		
2–5	26 (29.5%)	271 (34.3%)		
≧ 5	6 (6.8%)	113 (14.3%)		
Clinical features				
Cough	85 (96.6%)	759 (96.1%)	0.056	1.000
Wheezing	25 (28.4%)	140 (17.7%)	5.926	0.015*
Tachypnea	15 (17.0%)	71 (9.0%)	5.819	0.016*
Pharyngeal hyperaemia	65 (73.9%)	613 (77.6%)	0.627	0.429
Rales	30 (34.1%)	214 (27.1%)	1.935	0.164
Fever	58 (65.9%)	506 (64.1%)	0.119	0.730
WBC, 10^9^/L	9.2 (6.9, 11.5)	8.5 (6.8, 10.8)	− 1.302	0.193
HGB, g/L	128.0 (116.0, 137.0)	127.0 (118.0, 137.0)	− 0.012	0.990
LDH, U/L	306.5 (271.8, 344.3)	291.0 (254.5, 335.0)	− 2.231	0.026*
AST, U/L	34.6 (28.0, 39.0)	32.2 (25.6, 39.1)	− 1.384	0.178
ALT, U/L	13.9 (9.9, 20.1)	14.1 (10.1, 21.9)	− 0.558	0.577
Hospitalization days	7.0 (5.0, 8.8)	6.0 (5.0, 8.0)	− 0.770	0.441
Severe pneumonia	10 (11.4%)	39 (4.9%)	6.207	0.023*

Measurement data were expressed as median (IQR), and data were compared by rank sum test; Categorical variables were expressed as number and percentage, proportion were compared by chi-squared

Fever: T ≧ 37.5℃ (axillary temperature)

Reference levels: WBC (4.4–11.9)109/L, HGB (112–149) g/L, LDH (120–250) U/L, AST (13–35) U/L, ALT (7–40) U/L

*Age, Wheezing, Tachypnea, LDH and Severe pneumonia were statistically significant between the two groups (*P* < 0.05)

HBoV was detected at a significantly higher rate in patients aged < 2 years (63.6%), followed by aged 2–5 (29.5%) and aged ≧ 5 years (6.8%) (*P* = 0.048). Cough, pharyngeal hyperaemia and fever were the most frequent symptoms in HBoV positive children (*P* > 0.05). In addition, significantly more patients in HBoV positive groups developed wheezing (*P* = 0.015) and tachypnea (*P* = 0.016). Of interest, we observed that lactate dehydrogenase (LDH) in HBoV positive patients was higher than that in negative patients for laboratory test, the difference was statistically significant (*P* = 0.026). However, no significant difference between groups was noted for rales, hospitalization days, white blood cells count (WBC), hemoglobin (HGB), alanine aminotransferase (ALT) and aspartate aminotransferase (AST). Meanwhile, for those patients with CAP infected with HBoV, significantly more developed severe pneumonia than in those without HBoV (*P* = 0.023).

### Co-infection with other viruses and bacterium

**Fig. 4:**
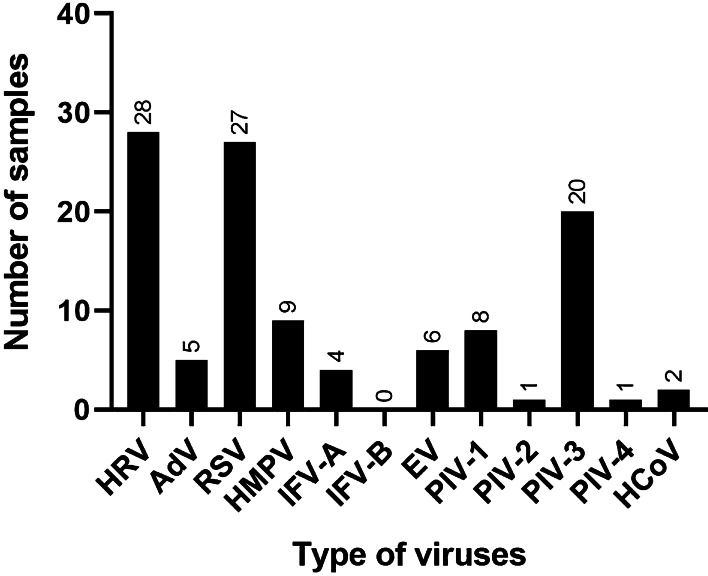
Co-infection distribution with HBoV

Among the patients who were positive for HBoV, HBoV alone was detected in 8 (9.1%), co-infection with two or more viruses in 76 (86.4%), with bacteria in 24 (27.3%), and co-infection with MP in 29 (33.0%), shown in Table 1. Of all 76 samples co-infected with other viruses, 47 samples with one type of virus detected, 24 samples with two type of viruses, 4 samples with 3 types of viruses, only 1 sample with four types of viruses (Table 3).The viruses most frequently co-infected with HBoV was HRV (n = 28), followed by RSV (n = 27), other viruses were shown in Fig. 4. In addition, there are 24 HBoV-positive samples co-infected with bacteria, as was showed in Table 4, 87.5% are gram negative bacteria, *Escherichia coli* were detected in 7 (29.2%) samples, which was dominant bacteria, *Klebsiella* pneumoniae was subordinate and in 5 (20.8%) samples.

**Table 3:** The type of HBoV co-infected with other viruses

	Type of co-infection	n (%)
Two types of viruses (n = 47, 53.4%)	HBoV + IFV-A	1 (1.1%)
	HBoV + RSV	15 (17.0%)
	HBoV + HRV	18 (20.5%)
	HBoV + HMPV	2 (2.3%)
	HBoV + PIV-1	2 (2.3%)
	HBoV + PIV-3	7 (8.0%)
	HBoV + EV	1 (1.1%)
	HBoV + AdV	1 (1.1%)
Three types of viruses (n = 24, 27.3%)	HBoV + HCoV + HMPV	1 (1.1%)
	HBoV + PIV-3 + RSV	4 (4.5%)
	HBoV + PIV-4 + EV	1 (1.1%)
	HBoV + RSV + IFV-A	1 (1.1%)
	HBoV + HCoV + RSV	1 (1.1%)
	HBoV + HRV + PIV-3	3 (3.4%)
	HBoV + AdV + PIV-3	1 (1.1%)
	HBoV + HRV + EV	2 (2.3%)
	HBoV + AdV + HMPV	1 (1.1%)
	HBoV + AdV + HRV	1 (1.1%)
	HBoV + RSV + HMPV	1 (1.1%)
	HBoV + IFV-A + HMPV	1 (1.1%)
	HBoV + IFV-A + RSV	1 (1.1%)
	HBoV + PIV-1 + RSV	2 (2.3%)
	HBoV + PIV-1 + HRV	1 (1.1%)
	HBoV + EV + PIV-3	1 (1.1%)
	HBoV + HMPV + PIV-3	1 (1.1%)
Four types of viruses (n = 4, 4.5%)	HBoV + AdV + HRV + PIV-1	1 (1.1%)
	HBoV + PIV-2 + PIV-3 + HRV	1 (1.1%)
	HBoV + PIV-1 + PIV-3 + RSV	1 (1.1%)
	HBoV + HMPV + EV + HRV	1 (1.1%)
Five types of viruses (n = 1, 1.1%)	HBoV + PIV-1 + PIV-3 + RSV + HMPV	1 (1.1%)

The percentage in the table represent the proportion of the subjects to the number of HBoV-positive cases (n = 88)

**Table 4:** Co-infection of HBoV with bacteria

Bacteria (n = 24)	n (%)
*Escherichia coli*	7 (29.2%)
*Klebsiella pneumoniae*	5 (20.8%)
*Enterobacter cloacae*	4 (4.5%)
*Streptococcal pneumonia*	2 (2.3%)
*Pseudomonas aeruginosa*	2 (2.3%)
*Bordetella bronchiseptica*	1 (1.1%)
*Staphylococcus aureus*	1 (1.1%)
*Enterobacter aerogenes*	2 (2.3%)
*Klebsiella oxytoca*	1 (1.1%)
*Stenotrophomonas maltophilia*	1 (1.1%)

### Clinical characteristics of HBoV-positive patients with or without bacteria

As was showed in Table 5, HBoV-positive patients less than 1 year old are more likely to co-infection with bacteria (*P* = 0.007), with the ratio of 66.7%. while there were no significant difference in the frequency and numerical value of other clinical features (gender, cough, wheezing, pharyngeal hyperaemia, rales, T≧38.5℃, severe pneumonia, WBC, HGB, AST, ALT, LDH, C-reactive protein, procalcitonin, neutrophilic granulocyte percentage and hospitalization duration) between the two group.

**Table 5:** Clinical characteristics of HBoV-positive patients with or without bacteria

Variable	With bacteria (n = 24)	Without bacteria (n = 64)	X^2^/Z	*P*
Gender			1.513	0.219
Male	18 (75.0%)	39 (60.9%)		
Female	6 (25.0%)	25 (39.1%)		
Age			14.245	0.007*
< 1	16 (66.7%)	16 (25.0%)		
1–2	5 (20.8%)	19 (29.7%)		
2–3	1 (4.2%)	9 (14.1%)		
3–5	1 (4.2%)	15 (23.4%)		
≧ 5	1 (4.2%)	5 (7.8%)		
Clinical Features				
Cough	22 (91.7%)	63 (98.4%)	2.430	0.179
Wheezing	9 (40.9%)	16 (27.6%)	1.318	0.251
Tachypnea	4 (16.7%)	11 (17.2%)	0.003	1.000
Pharyngeal hyperaemia	20 (83.3%)	45 (71.4%)	1.304	0.408
Rales	11 (45.8%)	19 (31.1%)	1.627	0.202
T ≧ 38.5℃	7 (29.2%)	25 (39.1%)	0.739	0.390
WBC, 10^9^/L	9.1 (7.1, 13.7)	9.2 (6.8, 11.4)	− 0.773	0.440
NEUT, %	46.4 (19.4, 66.5)	38.8 (30.3, 61.5)	− 0.473	0.636
LDH, U/L	312.5 (284.3, 363.0)	304.0 (270.3, 342.5)	− 0.947	0.344
AST, U/L	36.9 (29.8, 43.6)	33.8 (27.5, 38.1)	− 1.474	0.140
ALT, U/L	18.3 (10.9, 24.9)	13.5 (9.2, 17.9)	− 1.964	0.050
CRP, mg/L	6.0 (1.0, 25.3)	3.5 (1.1, 8.7)	− 1.010	0.312
PCT, ng/mL	0.29 (0.1, 3.7)	0.31 (0.11, 5.8)	− 0.972	0.331
Hospitalization days	7.0 (5.0, 9.0)	6.0 (5.0–8.0)	− 0.826	0.409
Severe pneumonia	4 (16.7%)	6 (9.4%)	0.921	0.451

Measurement data were expressed as median (IQR), and data were compared by rank sum test; Categorical variables were expressed as number and percentage, proportion were compared by chi-squared

CRP, C-reactive protein; PCT, procalcitonin

Reference levels: WBC (4.4–11.9)109/L, NEUT (40–75) %, LDH (120–250) U/L, AST (13–35) U/L, ALT (7–40) U/L, CRP (0–10) mg/L, PCT (0–0.046) ng/mL

*Age was statistically significant between the two groups (*P* < 0.05)

### Clinical characteristics of HBoV-positive patients with or without MP

Of all HBoV-positive patients, 29 (33.0%) co-infected with MP. Rales and fever were the more frequent in HBoV-positive patients co-infected with MP than without MP, but there were no statistical significance difference. Higher level of AST was found in HBoV-positive patients without MP (*P* = 0.004). In Table 6, data were compared between two groups, we found that the difference had no statistical for other clinical features.

**Table 6:** Clinical characteristics of HBoV-positive patients with or without MP

Variable	With MP (n = 29)	Without MP (n = 59)	X^2^/Z	*P*
Gender			1.107	0.293
Male	21 (72.4%)	36 (61.0%)		
Female	8 (27.6%)	23 (39.0%)		
Age			3.313	0.191
< 2	17 (58.6%)	39 (66.1%)		
2–5	8 (27.6%)	18 (30.5%)		
≧ 5	4 (13.8%)	2 (3.4%)		
Clinical features				
Cough	27 (93.1%)	58 (98.3%)	1.598	0.252
Wheezing	5 (20.8%)	20 (35.7%)	1.732	0.292
Tachypnea	4 (13.8%)	11 (18.6%)	0.324	0.765
Rales	14 (48.3%)	16 (28.6%)	3.248	0.071
Fever	18 (72.0%)	35 (60.3%)	1.028	0.455
WBC, 10^9^/L	7.6 (6.6, 10.5)	10.0 (7.4, 12.0)	− 1.633	0.102
CRP, mg/L	3.8 (1.8, 16.5)	3.9 (0.94, 11.2)	− 0.284	0.776
LDH, U/L	302.0 (270.0, 338.0)	311.0 (273.0, 345.0)	− 0.705	0.480
AST, U/L	31.3 (26.5, 35.3)	36.1 (30.0, 40.8)	− 2.857	0.004*
ALT, U/L	12.8 (8.7, 16.7)	15.6 (10.4, 21.2)	− 1.786	0.074
Hospitalization days	7.0 (5.0, 8.8)	6.0 (5.0, 8.0)	− 0.171	0.864
Severe pneumonia	2 (6.9%)	8 (13.8%)	0.857	0.487

Measurement data were expressed as median (IQR), and data were compared by rank sum test; Categorical variables were expressed as number and percentage, proportion were compared by chi-squared

Reference levels: WBC (4.4–11.9)109/L, CRP (0–10) mg/L, LDH (120–250) U/L, AST (13–35) U/L, ALT (7–40) U/L

*AST was statistically significant between the two groups (*P* < 0.05)

### Phylogenetic analysis

**Fig. 5:**
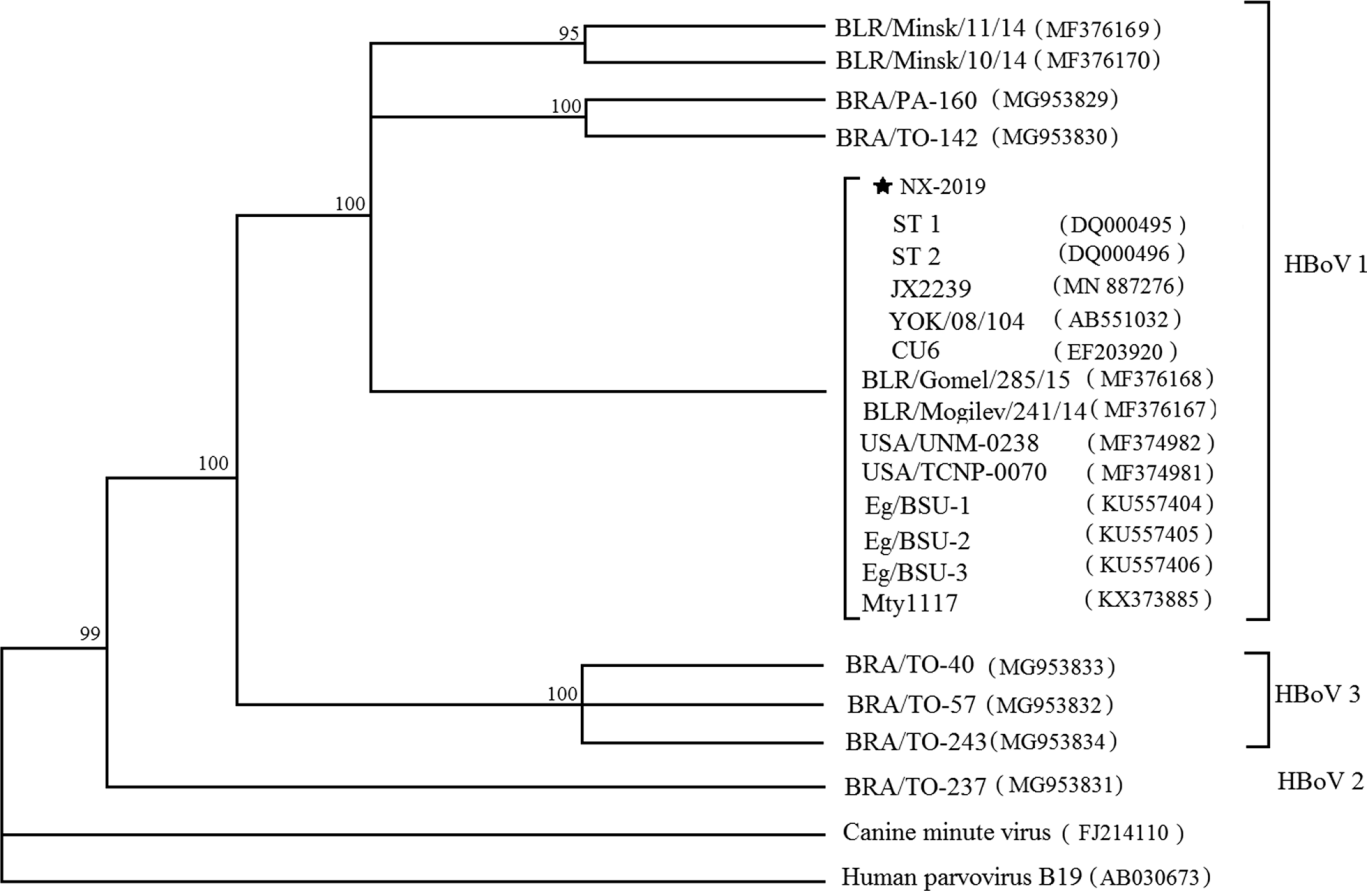
Phylogenetic analysis of the partial NS1 nucleotide sequences of HBoV. Phylogenetic tree with 1,000 bootstrap replicates was generated using the maximum likelihood method with MEGA 7.0 software. The number adjacent to the node represents the bootstrap value. HBoV sequences marked with pentagram was generated from the present study, and other reference sequences were obtained from GenBank

The partial fragments of NS1 (600 bp) of 8 positive specimens for HBoV were selected and sequenced, we found that the sequences of those specimens were consistent. HBoV strains used in the phylogenetic analysis included the strains obtained in our study in Ningxia, representative strains of HBoV-1, 2 and 3, canine minute virus and human parvovirus B19. Based on phylogenetic analysis (Fig. 5), HBoV strains in this research clustered with the HBoV-1 genotype, and sequences were genetically related (nucleotide identity was 100%) to previous strains detected in Sweden (DQ000495, DQ000496), China (MN887276), Japan (AB551032), Thailand (EF203920), United States (MF374981, MF374982) and other strains showed in Fig. 5.

## Discussion

In this study, we used multiplex PCR to detect HBoV and other viruses in hospitalized CAP children under 14 years old in Ningxia, China. In all 878 cases, virus was detected in the highest rate, which is consistent with previous studies [1, 13, 14], followed by bacteria, and the lowest was MP, certainly, different age groups have different susceptibility to pathogens. The number of HBoV-positive cases were 88 (10.0%) in our study, and the detection rate of HBoV varies in different countries, which is related to different regions, genetic and detection methods. As previously reported [15], the worldwide average prevalence of HBoV in respiratory tract samples ranged from 1.0% (CI 0.0–2.0) to 56.8% (CI 46.9–66.8), and the total prevalence estimates in respiratory infections was 6.3% (CI 6.2–6.4).The other common viruses in our research are HRV and RSV, this is in line with previous studies [16, 17].

The seasonal peaks of HBoV infections vary among countries because of differences in climatic, geographic factors and population enrolled. In this study, HBoV activity peaked in winter, followed by autumn, it was possibly due to the dry, cold weather, and in agreement with the previous report [18, 19]. In contrast, the seasonal prevalence of HBoV in Changsha and Guangzhou peaked in summer [10, 20], perhaps, hot and humid weather was responsible for that.

In our study, HBoV infected children aged less than 1 month to 13 years 5 months with CAP, the most typical age group was less than 2-years-old, which indicated that infants with lower immunity are more likely to be infected with HBoV. Other studies have also reported HBoV infection is more common in children under two years old, and only rarely has been found in adults and the elderly age [21, 22]. With respect to the clinical features of HBoV infection, our research demonstrated that cough, pharyngeal hyperaemia and fever were the most frequently observed, which were also commonly seen among CAP without HBoV. Thus, clinically, it is impossible to differentiate patients with pneumonia caused by HBoV infection or other pathogens from those symptoms. Interestingly, patients infected with HBoV are more likely to cause wheezing, and it is already known that viral infection could trigger airway hyperresponsiveness and inflammatory immune response, which leading to wheezing, these reactions may be more susceptible to occur after HBoV infection, association of it in respiratory samples and wheezing has been also described in other researches [23–25]. Furthermore, significantly more patients in HBoV positive groups developed tachypnea, this was possibly due to the high incidence of wheezing. We also observed LDH was higher in HBoV-positive patients, which was universally elevated in many pulmonary diseases and has been reported in several studies to be associated with disease severity [26, 27]. Indeed, our data suggest that HBoV-positive patients with CAP were more likely to progress into severe pneumonia, the result was consistent with other studies [28, 29]. Researcher have pointed out that HBoV-1 infection in polarized primary human airway epithelia (HAE) caused airway epithelial damage, including disruption of the tight junction barrier, loss of cilia, and epithelial cell hypertrophy [30]. And among all the HBoV-positive patients, 8 cases were infected alone. Taken together, HBoV may be one of the pathogens for infants and young children with CAP. Other published reports also implied that there is a significant association between HBoV infection and CAP in children [31–33].

HBoV mono-infection is rare, co-infection was observed frequently. There were no significant differences in clinical characteristics between patients with HBoV infection alone and patients with co-infection in our study. Of all the cases infected with the HBoV, 90.9% were co-infections with other pathogen. By far, few published reports demonstrated detailed type of co-infection of HBoV with other viruses and bacterium for CAP in children, in this study, we counted all the types of viruses and bacteria co-infected with HBoV, so as to provide more reference information for clinical work. 86.4% of the HBoV-positive patients co-infected with other respiratory viruses, as showed in Table 3, double infection was the most common, accounting for 53.4%, we even detected four types of viruses co-infected with HBoV in respiratory secretions. Of all co-infected viruses, the top three were HRV, RSV and PIV-3, previous reports showed the most frequently co-infected were RSV, HRV, HMPV and PIV-3 [31, 34, 35], our data were in line with it. Bacteria was detected in 27.3% of HBoV-positive patients, and *Escherichia coli*, *Klebsiella pneumoniae* and *Enterobacter cloacae* were principal types of bacteria detected in HBoV-positive patients,87.5% are gram negative bacteria, and the bacterium spectrum was consistent with those in bacterial pneumonia in children. HBoV has been detected in children with or without respiratory symptoms [36–39], and it was found in some asymptomatic children undergoing elective surgery and tonsillectomy [40, 41], which suggest that there is a latent or persistent infections of HBoV in respiratory tract, and a study demonstrated HBoV-1 is often detectable with other viruses in asymptomatic patients, promoting the reactivation of a latent virus by a super-infection [42], all of the above may explain the high prevalence of co-infections.

In our study, dozens of HBoV-positive cases co-infected with bacteria, so we analyzed the clinical characteristics of HBoV-positive patients cases with or without bacteria. In consideration of the age distribution between two groups, HBoV-positive patients less than 1 year old are more susceptible to bacterial infection. In agreement with previous study [43], no significant differences were found in term of frequencies of cough, wheezing and number of hospitalization days. Besides, other clinical features in Table 5 with no statistical significance. Higher proportion of patients with wheezing, rales and severe pneumonia, and higher levels of LDH, AST, ALT and CRP were found in patients with HBoV co-infected with bacteria. Though their statistical significance might had been limited by small sample size, still these might suggest bacterial co-infection would trigger exacerbation of pneumonia.

In recent years, MP showed a yearly upward trend in the detection of pneumonia, this study reported the frequency of MP in pediatric patients diagnosed with CAP was identified in 21.0%, which was similar to several reports [44, 45], however, other studies report lower frequencies [46, 47]. 33.0% of HBoV-positive patients co-infected with MP in our research, compared with the other group who did not co-infected with MP in HBoV infection children, lower level of AST was found, but a study showed that *Mycoplasma pneumoniae* pneumonia patients in the acute phase had higher levels of AST than those of the patients in the recovery patients and healthy children [48]. It was also reported that AST level of patients with *Chlamydia* pneumonia was higher than that of *Mycoplasma* pneumonia [49]. In our study, MP is not the single pathogenic agent of CAP, and viral infection can also increase AST level. In addition, existing laboratory testing techniques are difficult to distinguish between MP carrier and infection status, so the basis for diagnosis of MP infection is still controversial [50], and the results of serum antibody may be false positive or false negative. So, we still need a lot of experiments to confirm this result. For other indicators in Table 6, there was no statistically significant differences between the two groups. These findings revealed that to distinguish CAP patients with HBoV co-infected with MP or not can rarely be established based on the clinical presentation alone.

We selected eight HBoV-positive samples, sequenced partial fragments of NS1, and found that the sequences of those specimens were consistent, which showed a high nucleotide identity between HBoV sequences, and phylogenetic analysis demonstrated that they belonged to HBoV-1. Similar results were revealed in previous studies [32, 51, 52].

This study has the following limitations. Firstly, we may have missed additional viruses not detected by current panels of PCR in our study. Secondly, all our study subjects were hospitalized patients with CAP, the results are not necessarily generalizable to outpatient clinics.

## Conclusions

Our study described the epidemiological and clinical features in HBoV-positive infants and children with CAP, and the findings highlighted the importance of HBoV infection in infants and children with CAP. The high incidence of severe pneumonia was found in HBoV-positive patients compared with HBoV-negetive cases may indicate a relationship between severe pneumonia and HBoV. Undeniably, there is a limited population of enrolled patients in this study, therefore further studies are required to investigate the relationship between HBoV infection and CAP in children.

## Data Availability

All data generated or analyzed during this study are included in this published article.

## Abbreviations

CAP: Community-acquired pneumonia
HBoV: Human bocavirus
MP: *Mycoplasma pneumoniae*
RSV: Respiratory syncytial virus
IFV: Influenza virus
HRV: Human rhinovirus
AdV: Adenoviruses
HCoV: Human coronaviruses
PIV: Parainfluenza viruses
HMPV: Human metapneumovirus
EV: Enterovirus
WBC: White blood cells count
HGB: Hemoglobin
AIL: Alanine aminotransferase
AST: Aspartate aminotransferase
CRP: C-reactive protein
PCT: Procalcitonin
